# Tyrosine Kinase Inhibitor Therapy Enhances Stem Cells Profile and May Contribute to Survival of Chronic Myeloid Leukemiastem Cells

**DOI:** 10.3390/jcm14020392

**Published:** 2025-01-10

**Authors:** Simone Rocco, Alessandro Maglione, Valentina Schiavo, Alessandro Ferrando, Carmen Fava, Daniela Cilloni, Barbara Pergolizzi, Cristina Panuzzo

**Affiliations:** Department of Clinical and Biological Sciences, University of Turin, 10124 Orbassano, Italy; simone.rocco@unito.it (S.R.); alessandro.maglione@unito.it (A.M.); valentina.schiavo@unito.it (V.S.); alessandro.ferrando@unito.it (A.F.); carmen.fava@unito.it (C.F.); daniela.cilloni@unito.it (D.C.); barbara.pergolizzi@unito.it (B.P.)

**Keywords:** SOX2, OCT 3/4, leukemic stem cells (LSC), TKI, resistance, ROS, glutathione, FAO

## Abstract

**Background/Objectives**: Treatment with tyrosine kinase inhibitors (TKIs) in chronic myeloid leukemia (CML) has revolutionized disease management and has transformed CML from a life-threatening disease to a chronic condition for many patients. However, overcoming resistance, particularly related to leukemic stem cells (LSC) that can persist even when the bulk of the leukemic cells are eliminated, remains a significant challenge. **Methods**: K562 and KU812 cell lines were treated in vitro with the TKI Imatinib (IM). Gene expression, protein analysis, and metabolomic screening were conducted to investigate the ability of the drug to enhance stem cell (SC) features. Moreover, a gene ontology analysis was performed on different available datasets, to further consolidate our data. **Results**: 48 h of IM treatment can significantly increase the expression of genes related to SC self-renewal, particularly SOX2 and OCT 3/4. Interestingly, these modulations occur in cells that remain alive after drug treatment and that displayed features consistent with leukemia stem-like CML cells, suggesting that SC genes levels are crucial even in cell population survived upon TKI treatment. Moreover, after in silico analysis of available data, we observed an enrichment of SOX2/NANOG and OCT 3/4 signatures after TKI treatment, thus strengthening our results. **Conclusions**: Our results confirmed the relevance of LSC features after TKI treatment, highlighting the need for more effective and potentially curative strategies targeting LSCs to overcome resistance in CML.

## 1. Introduction

Chronic myeloid leukemia (CML) is a myeloproliferative disorder characterized by a significant expansion of primitive hematopoietic cells [1]. CML is the first human pathology identified to involve a specific karyotype abnormality, the Philadelphia chromosome (Ph) [2]. The detection of the Ph chromosome is currently used for diagnosing CML, molecular monitoring of residual disease, and follow up. The Ph chromosome results from a genetic translocation between chromosome 22 and chromosome 9, designated t (9;22), involving BCR and c-ABL genes, respectively [3,4]. The resulting BCR-ABL fusion protein exhibits oncogenic features, primarily due to its constitutive tyrosine kinase activity. These events lead to the transformation of hematopoietic stem cells and development of leukemic cells [5,6,7].

The functional significance of BCR-ABL, along with its oncogenic tyrosine kinase (PTK) activity, enabled the approval of the first generation of tyrosine kinase inhibitors (TKI), Imatinib (IM, Glivec, Novartis, Basel, Switzerland), in 2001. IM revolutionized the treatment of CML, given its ability to directly target the BCR-ABL enzymatic activity [8].

Second-generation TKIs such as Nilotinib (Tasigna; Novartis, Basel, Switzerland) and Dasatinib (Sprycel; Bristol–Myers Squibb, New York, NY, USA) were subsequently approved to treat patients resistant or intolerant to IM, as this class of inhibitors can bind and block 14 of 15 different IM-resistant isoforms of BCR-ABL [9]. Third generation TKIs like Ponatinib ((Iclusig), Takeda Pharmaceuticals U.S.A., Inc., Cambridge, MA, USA) were developed to treat adult CML patients resistant to other TKIs, particularly those harboring T315I mutation [10]. The use of TKIs as treatment led to the achievement of complete cytogenetic remission and major molecular response (CCgR and MMR) in the majority of CML cases. As a result, TKIs discontinuation has become a primary objective in the clinical management. However, resistance mechanisms, including BCR-ABL amplification, BCR-ABL mutations, and leukemic clonal evolution, remain a challenge [11,12]. Notably, the persistence of a primitive population of leukemia stem cells (LSCs) that can escape TKI treatment is a major cause of TKI resistance. LSCs possess self-regenerative capabilities, contributing to cancer expansion and driving disease relapse and progression [13].

McCulloch and Till characterized stem cells (SC) by three specific properties: self-renewal, differentiation, and proliferative capacity [14]. Key molecular regulators of SC-specific functions include POU5F1 (alias OCT 3/4) and SOX2, which cooperate with other transcription factors to support the expression of pluripotency genes essential for stemness. Dysregulation of these genes triggers cascades that contribute to cancer pathogenesis, including downregulation of tumor suppressors, induction of therapy resistance, and relapse [15,16,17].

Recent studies have highlighted the role of metabolic pathways alterations following TKI treatment in promoting LSC resistance and poor prognosis [18,19]. In particular, pathways related to glucose/glutamate metabolism, amino acid metabolism and lipid metabolism are significantly upregulated, suggesting a LSC adaptation in order to survive [20,21].

In this context, there is an urgent need to better discern the mechanisms sustaining LSC maintenance and survival to minimize relapse and improve long-term outcomes. Therefore, the aim of our project was to investigate whether genes related to SC features, along with metabolic changes induced by drug treatment, contribute to TKI resistance. Using a CML cell line model, complemented by in silico analysis of available datasets, we described the unique association between specific SC-related genes and TKI treatment. These findings suggest potential novel approaches for molecular monitoring of CML progression.

## 2. Materials and Methods

### 2.1. Cells Line Treatment

The K562 and KU812 cell lines were purchased from American Type Culture Collection (ATCC, Manassas, VA, USA), grown in RPMI 1640 medium supplemented with L Glutamine and sodium bicarbonate (Sigma-Aldrich, St. Louis, MO, USA), 10% inactivated fetal bovine serum (FBS, Sigma-Aldrich, St. Louis, MO, USA), and 0.1% penicillin/streptomycin (EuroClone, Milan, Italy), and maintained at 37 °C with 5% of CO_2_. To isolate living cells from dead ones, a Ficoll treatment using Lymphocyte Separation Media (Lymphosep, AUROGENE, Rome, Italy) was performed according to the manufacturer’s instructions. Live cells were collected separately and washed with PBS 1X through centrifugation at 1300 rpm × 5 min.

### 2.2. Proliferation Assay

Cells growth was evaluated by MTT assay (Cell Proliferation Kit I (MTT) Roche Diagnostics, Indianapolis, IN, USA) according to the manufacturer’s instructions. We set four different conditions of IM treatment (0, 0.5, 1, 2 µM). To begin, 30,000 cells/per well were seeded in triplicate for each condition in a 96-well plate. After 48 h of treatment, MTT reagent was added inside each well and, after appropriate incubation, the absorbance at 550–600 nm was measured.

### 2.3. Western Blot

K562 and KU812 cells were treated with different concentrations of IM (0, 0.5, 1, 2 µM) for 48 h. Subsequently, cells were collected and lysed with RIPA buffer for 20 min. After a centrifugation at 4 °C, at 12,000 rpm for 10 min, the supernatants were quantified using the Bradford method and 30 µg of proteins were loaded, and then resolved through SDS-PAGE 8% for 1.30 h at constant 130 V. Subsequently, gels were transferred on PVDF membrane (Amersham Hybond, Sigma-Aldrich, St. Louis, MO, USA) for 1 h at constant 400 mA. The membranes were saturated with skimmed milk 5% for 2 h at 37 °C and incubated overnight at 4 °C with primary antibodies of interest (p-Tyr: Sc- 7020, Actin: Sc-4778, BCR: sc-365728, Santa Cruz Biotechnology, Dallas, TX, USA). After appropriate secondary antibodies incubation (Anti-rabbit: MES176703, Anti-mouse: MBS176647, MyBioSource, San Diego, CA, USA), immuno-reactive bands were observed at Chemidoc by using chemiluminescent enhanced reagent (Clarity Western ECL Substrate #170-5061, Bio-Rad, Hercules, CA, USA) and analyzed using the Image Lab program 6.0.0 2017 (BioRad Laboratories, Hercules, CA, USA).

### 2.4. RNA Extraction and qRT-PCR Analysis

The RNA was extracted using TRI Reagent (Sigma, St. Louis, MO, USA) according to the manufacturer’s instructions. All samples were quantified using Nanodrop (ThermoFisher, Waltham, MA, USA) and 500 ng of total RNA was used as template for the reverse transcription reaction with High-Capacity cDNA Reverse Transcription Kit (Applied Biosystem, Foster City, CA, USA) following the specified protocol. Expression level of Sox2 (Hs01053049_s1) and OCT3/4 (Hs0999634_gH) were investigated with TaqMan technology using a SensiFAST Probe No-ROX kit (Meridian Bioscience, Milan, Italy), through the C1000 Thermal Cycler CFX96 Real-Time System (Bio-Rad, Hercules, CA, USA). qRT-PCR data were analyzed by Bio-Rad CFX Manager 3.1 software (Bio-Rad, Hercules, CA, USA). The analysis was performed in triplicate and gene expression was expressed after normalization with GusB (Hs00939627_m1) housekeeping gene.

### 2.5. Immunofluorescence

Immunofluorescence was performed after executing a cytospin of 50,000 cells [22]. Cells were fixed with 4% paraformaldehyde (PFA) (Sigma-Aldrich, St. Louis, MO, USA), quenched with NH4Cl for 20 min, then subjected to cell permeabilization with 0.1% of Triton, and then saturated with blocking solution (PBS, 1% BSA, 0.3 M glycine) for 40 min. Subsequently, slides were incubated for 2 h with primary antibodies of interest (Santa Cruz Biotechnology, Dallas, TX, USA). Alexa Fluor 488 secondary antibodies (Invitrogen, Waltham, MA, USA) were added for 40 min for protein detection. Slices were mounted with Vectashield Mounting Medium with DAPI (Vector Laboratories, Newark, CA, USA) and visualized with a confocal microscope (LSM 800; Carl Zeiss MicroImaging Inc., Oberkochen, Germany) using a 63X objective. Figures were analyzed and quantified using Fiji 2 software (https://imagej.net/software/fiji/downloads, accessed on 28 December 2024).

### 2.6. Apoptosis Assay

Apoptosis assay was performed using cells after Ficoll treatment and evaluated by flow cytometry (FACS) [23]. Briefly, cells were washed with PBS 1X and incubated in Annexin buffer 1X with FITC-conjugated annexin V and propidium iodide (PI) (Biolegend, San Diego, CA, USA). BD FACSDiva v9.0 Software (BD Biosciences, Franklin Lakes, NJ, USA) was used for data analysis of Annexin V-positive cells.

### 2.7. ROS Measurement

The ROS level was measured with the ROS-sensitive fluorescent probe 5-(and-6)-chloromethyl-2′,7′-dichlorodihydro-fluorescein diacetate-acetoxymethyl ester (DCFDA-AM) [22]. The results are expressed as nmol/mg.

### 2.8. Samples Preparation for HPLC-HRMS Analysis

Pellets derived from five million cells under different experimental conditions were washed twice with PBS buffer. The dry pellet was resuspended in 250 µL of MilliQ water and lysed by alternating cold (liquid nitrogen) and hot (45 °C) baths. Subsequently, 250 µL of acetonitrile (ACN) with 1% formic acid was added, followed by vortexing for 2 min. The mixture was then stored at −20 °C for 30 min to facilitate protein precipitation. After centrifugation at 13,000 rpm for 10 min, 200 µL of the supernatant was collected and transferred to a vial for HPLC-MS analysis.

### 2.9. HPLC-HRMS Metabolomic Analysis

To investigate the molecular composition of these samples, an HPLC-HRMS analysis, through HPLC-timsTOF Pro 2 instrument (Bruker Daltonics GmBH & Co., Bremen, Germany), was conducted. The HPLC was equipped with a Luna Polar C18 column (2.1 × 150 mm, 3 μm) (Phenomenex, Bologna, Italy) and the system was operated in binary gradient mode. Water at 0.1% of formic acid was used as solvent A and ACN at 0.1% of formic acid as solvent B. The chromatographic gradient was set as follows: flow, 0.3 mL/min; t: 0 min B: 5%; t: 15 min B: 100%; t: 16 min B: 100%; column reconditioning was conducted for 4 min at B: 5%. HRMS instrument was coupled to HPLC with a VIP-HESI source with the following parameters both in positive and negative ion modes: End Plate Offset of 500 V, Capillary Voltage of 4.5 kV; Nebulizer and Dry gas flow rate were, respectively, 2 bar and 8 L/min; Dry and Sheath gas temperature were, respectively, 230 °C and 400 °C. The mass spectrometer was operated in full-scan mode in the range 20–1300 *m*/*z*, with a resolution of 30,000 in FTMS. DDA tandem mass experiments were performed in the range 20–1300 m/z for both polarities. Auto MS/MS was used to collect the MS/MS spectra with a spectra dynamic control of spectra rate from 16 to 20 Hz. Collision energy was set at 20 eV for the range of 50–1000 *m*/*z* and at 30 eV for 1300 *m*/*z*. All spectra were acquired in profile mode. MetaboScape 2023b software (Bruker Daltonics GmBH & Co., Bremen, Germany) was used for data elaboration and calculation.

### 2.10. Data Pre-Processing and Analysis

A MetaboScape principal component analysis (PCA) was performed to assess the statistical stability and separation of the data groups; then the features table was filtered to include only the annotated ones. MetaboAnalyst 6.0 (https://www.metaboanalyst.ca/MetaboAnalyst/ModuleView.xhtml, accessed on 28 December 2024) was subsequently used to identify significant factors and to highlight the metabolites with differential expressions. From these analyses, a heatmap was generated to visualize the expression patterns of the metabolites, and pathway enrichment analysis was conducted to identify key biological pathways associated with the observed metabolic changes.

### 2.11. Gene Expression Analysis of CML Models

Gene expression profiling of Imatinib-treated K562 cells or CD34+ derived from CML cells were retrieved from GEO (GSE120932 and GSE198576) [24,25]. In the first dataset, K562-IR (K562-derived drug-resistant sub-clone) samples (GSM3421755, GSM3421756, GSM3421757) treated with 10µM imatinib and untreated standard K562 cell line samples (GSM3421746, GSM3421747, GSM3421748) were analyzed by the GEO2R tool. In the second dataset, CD34+ derived from CML patients at diagnosis (GSM5952552, GSM5952547, GSM5952542) or treated in vitro with IM for 72 h (GSM5952545, GSM5952550, GSM5952540) were analyzed by the GEO2R tool. Identified significant modulated genes obtained by GEO2R (Log2FC > 2 or <−2 and FDR adjusted *p*-value < 0.001 for GSE120932, Log2FC > 1 or <−1 and FDR adjusted *p*-value < 0.05) were submitted to gene ontology analysis using the tool EnrichR (https://maayanlab.cloud/Enrichr/, accessed on 28 December 2024) [26]. The first 10 enriched terms from the ChEA Transcription Factor Targets Dataset (ChEA) 2022 were ordered by *p*-value and reported using the Appyter web-tool integrated in EnrichR.

### 2.12. Statistical Analysis

Statistical analysis was performed using the paired t-test. All the experiments were performed in triplicate and significance was marked as follows: * *p* ≤ 0.05, ** *p* ≤ 0.01, and *** *p* ≤ 0.001.

## 3. Results

### 3.1. Imatinib Treatment Induces a Significant Increase of Genes Related to Stemness

**Figure 1:**
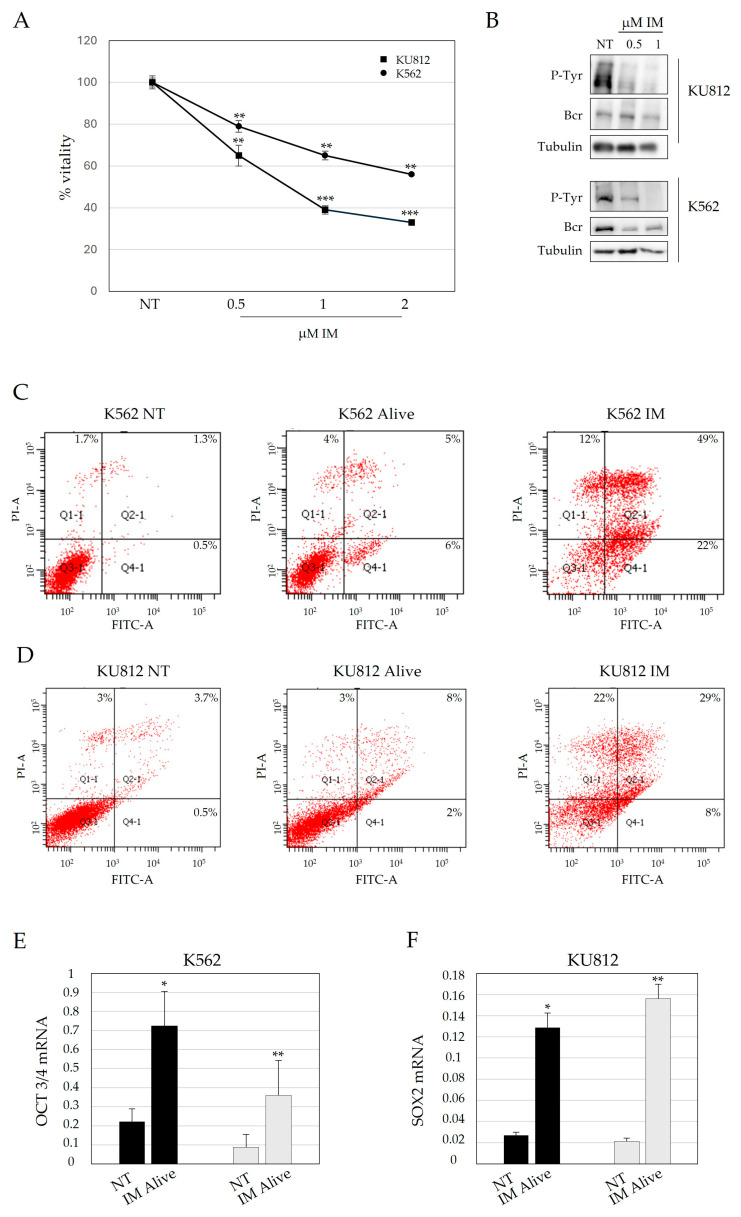
Imatinib treatment induces a significant increase of genes related to stemness. (**A**) K562 and KU182 cell lines were treated with different concentrations of IM (0, 0.5, 1, 2 μM) to evaluate cell proliferation rate. Not treated cells are considered 100% vitality. (**B**) Western blot analysis of BCR/ABL phosphorylation level demonstrates its decrease in a dose dependent manner, confirming the activity of TKI. (**C**,**D**) FITC-Annexin V and PI staining was performed to confirm by FACS analysis the vitality of cells after Ficoll stratification. (**E**,**F**) OCT3/4 and SOX2 gene expression were evaluated through RT-qPCR, after 48 h of IM treatment cells extracted with Ficoll. Abbreviations: NT: not treated; IM: Imatinib; Ann V, Annexin V. * *p* ≤ 0.05, ** *p* ≤ 0.01, *** *p* ≤ 0.001.

Imatinib treatment completely revolutionized the prognosis of CML even if it does not eradicate LSC. We treated K562 and KU812 with different concentrations of drug (0.5, 1, and 2 µM) for 48 h to evaluate the modulation of stem cell (SC)-related genes that may be responsible for IM resistance. IM reduces the proliferation of K562 and KU812 in a dose dependent manner and causes a strong decrease in BCR/ABL phosphorylation level, as demonstrated by MTT assay and Western blot, respectively (Figure 1A,B). Subsequently, we decided to investigate the expression of genes related to SC self-renewal, thus SOX2 and OCT 3/4 on cells extracted after Ficoll density gradient centrifugation. By using this method, we can isolate the fraction of cells that survived after IM treatment, eliminating dead cells that might interfere with gene expression quantification.

After confirming that cells collected after Ficoll stratification are not under significant apoptosis, as shown in Figure 1C,D, we proceeded with RNA extraction and gene expression analysis. Figure 1E,F confirmed that in the fraction of alive K562 and KU812, SOX2 and OCT 3/4 levels increased significantly after treatment (IM alive), suggesting that SC gene levels are crucial even in cells unaffected by TKIs.

### 3.2. Imatinib Treatment Induces Nuclear Localization of Both Sox2 and OCT 3/4 and Is Associated with an Increase in the LSC Phenotype

**Figure 2:**
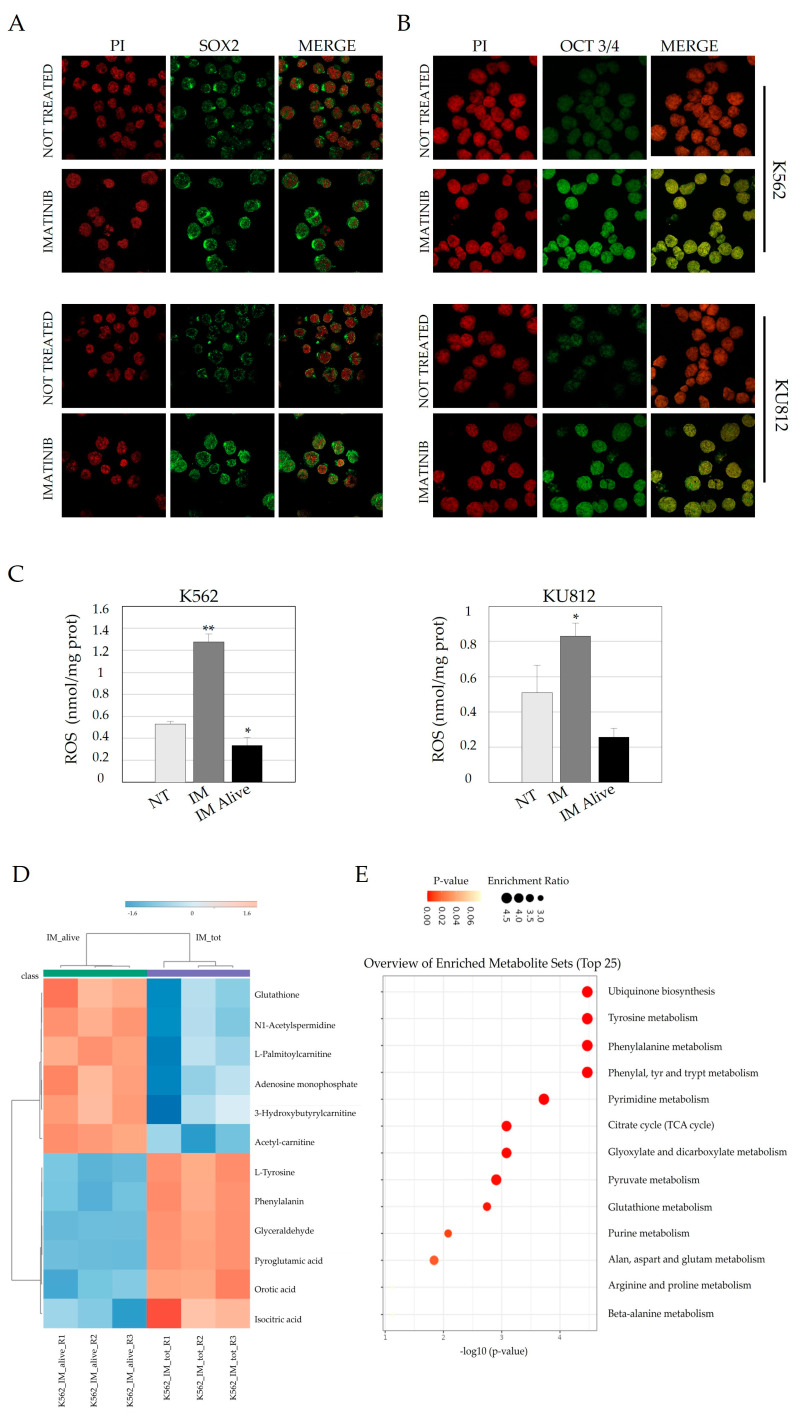
Imatinib treatment increases protein levels of SOX2 in K562 and KU812. (**A**,**B**) The green signal corresponding to Sox2 or OCT 3/4 showed a strong nuclear increase after IM treatment in alive cells extracted with Ficoll gradient. Red propidium (PI) is used to detect nuclei (63X magnification). (**C**) ROS levels in cytosolic extract of cells treated with IM, ROS is expressed as nmol/mg of proteins. (**D**) Heatmap of the most significant expressed metabolites in the groups of alive and total cells after Imatinib treatment. Significantly reduced activities are represented in blue while those with a significantly increased activity are in red. K562 IM alive R1, 2, and 3 corresponded to viable cells condition triplicates while K562 IM tot R1, 2, and 3 represented the total cells after treatment condition triplicates. (**E**) Pathway enrichment analysis obtained with MetaboAnalyst and KEGG for pathway identification. The significance of a particular pathway was expressed as enrichment ratio. The *p*-value is represented with a red scale. * *p* ≤ 0.05, ** *p* ≤ 0.01.

Since the mRNAs of the investigated genes related to stem cells increased after IM treatment in the fraction of viable cells, we decided to assess the levels and localization of the corresponding proteins through immunofluorescence analysis. Sox2 exhibited predominant nuclear localization, with its levels significantly increasing after 48 h of 1 µM IM treatment, as indicated by a sharp green nuclear accumulation (Figure 2A) in both the cell lines analyzed. A similar pattern was observed for OCT 3/4 (Figure 2B), which displayed a significant increase in nuclear localization with a spotted distribution, in accordance with its ability to form a complex with Sox2 and co-bind to specific DNA sequences.

Overall, these results confirm the activation of transcription factors in the viable pool of cells, a process that could be essential for inducing the expression of crucial genes in the stemness compartment. Subsequently, since ROS levels are crucial to define an increase of stem characteristic, we measured it in cells treated with IM for 48 h and in alive cells collected after treatment. ROS levels were significantly reduced in cytosolic extracts of viable cells compared to the total amount of treated cells (Figure 1E). This result is important since low ROS levels are currently used as a parameter to isolate LSC, thus supporting the hypothesis that our population of cells surviving IM treatment might acquire specific features resembling those of resistant stem cells [27].

Subsequently, to investigate whether the pool of viable cells exhibited additional signatures resembling that of stem cells, we performed an untargeted metabolomic analysis using the HPLC-timsTOF Pro 2 instrument (Bruker Daltonics GmBH & Co., Bremen, Germany). The resulting metabolites were analyzed using MetaboAnalyst to identify those significantly expressed.

After conducting an ANOVA test between the two groups, the resulting heatmap revealed sharp clustering, suggesting distinct metabolic features between the two groups. Interestingly, glutathione emerged as one of the most important metabolites in the pool of viable cells, alongside L-Palmitoylcarnitine, 3-Hydroxybutyrylcarnitine, and adenosine monophosphate (AMP). Their roles in protecting cells from oxidative stress, maintaining low ROS levels, fueling energy demands, and preserving the cellular metabolic and energetic homeostasis are well-known in LSCs.

Finally, pathway enrichment analysis was conducted with MetaboAnalyst and KEGG to identify key biological pathways associated with the observed metabolic changes. Interestingly, the identification of pathways related to redox homeostasis, energy production through fatty acid oxidation, amino acid metabolism, and oxidative metabolism in the viable cells strengthened our findings.

### 3.3. Imatinib Treatment Activate a Few SC Transcription Factors in Cell Lines and in CD34+ CML Cells After In Vitro Treatment

**Figure 3:**
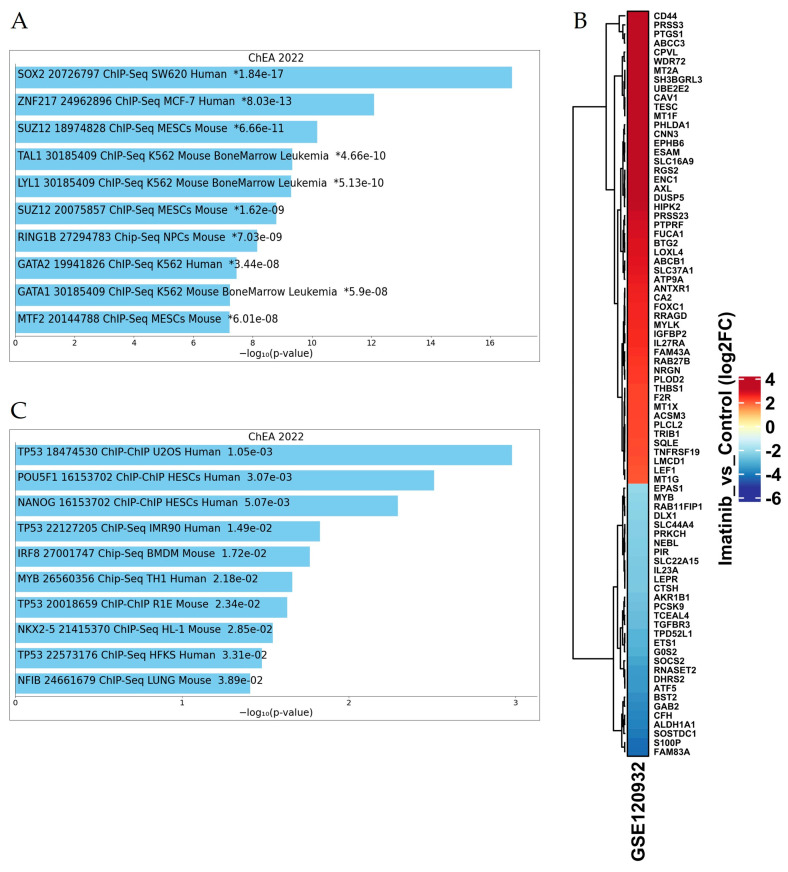
Imatinib treatment activate few SC transcription factors in cell lines and in CD34+ CML cells after in vitro treatment. (**A**) Differentially expressed genes among K562 sensitive and resistant to IM clusters enriched according to Gene Ontology terms and ordered by adjusted *p*-value. (**B**) Heatmap of all genes modulated by Sox2 emerged after comparison between control and Imatinib treatment K562 cells. (**C**) Differentially expressed genes after CD34+ CML cells treatment in vitro with IM clusters enriched according to Gene Ontology terms and ordered by adjusted *p*-value. * adjusted *p*-value.

To further strengthen our findings, we decided to evaluate a potential involvement of SC genes in cells resistant to TKI treatment by analyzing a public dataset comparing two groups: K562 cells sensitive and resistant to IM [28]. We investigated the pathway enrichment by EnrichR and ChEA 2022 as source by using all the 313 genes that emerged significantly deregulated. Notably, Sox2 was identified as the top transcription factor activated in the resistant cell group (Figure 3A), as it regulates 82 out of the 313 detected genes (Figure 3B). The EnrichR analysis of these differentially expressed genes highlighted oxidative stress and redox pathway as among the most affected, alongside focal adhesion and TGF-beta signaling. Interestingly, CD44 and Cav-1, emerging among the most significantly highly expressed, are known to promote quiescence of LSCs and can support cell cycle progression through G2/M phase when deleted, thus enforcing our analysis [29,30].

In addition, several crucial stem cell factors, including OCT 3/4 and Nanog, emerged in another re-analysis of public dataset of CD34+ derived from CML at diagnosis and treated in vitro with IM for 72 h. Even if the number of upregulated genes is reduced compared to cell lines specimens, these findings reinforce the link between these pathways and TKI treatment in a clinical context, thereby corroborating our results (Figure 3C) [25].

## 4. Discussion

LSCs are a subpopulation of cells with distinct features, including unique cell surfaces antigens, molecular pathways, and metabolism that support their expansions [13,31]. Moreover, these cells have a remarkable ability to resist elimination by standard TKI treatments and may serve as potential source for disease relapse [32]. This represents the most significant challenge in current leukemia treatment. In this context, data from multiple clinical trials suggest that tyrosine kinase inhibitor therapy can be discontinued in approximately half of CML patients who achieve a deep molecular response, following a program known as therapy-free remission (TFR) [33,34]. During this time, the probability of LSC emergence and an increase in the bulk of leukemia cells is high. Several studies have confirmed the persistence of LCS using highly sensitive technique like digital polymerase chain reaction (ddPCR), suggesting that even if patients remain in TFR or have activated a program of TKI discontinuation, a quote of resilient LSCs can persist for a long time [34,35]. These studies highlight the complexity of LSCs and their resistance to TKI. Understanding the molecular processes involved in LSCs and in their resistance to Imatinib has been the focus of several studies in the last decades, and it will be crucial for developing more effective targeted therapies and improving outcomes for leukemia patients in the near future.

In our study, we observed that after in vitro treatment of K562 and KU812, commonly used CML cell lines, the expression of certain key genes, specificallySox2 and OCT 3/4, was significantly increased. Interestingly, this increase was particularly noticeable in the fractions of samples that remain alive after treatment, suggesting that TKIs may activate a program aimed at maintaining the stem cells characteristics. Moreover, the sharp nuclear localization of Sox2 and OCT 3/4 confirmed the activation of these transcription factors, indicating that they could act as a source of disease relapse.

These genes play a significant role in SC maintenance and self-renewal, and their involvement in resistance mechanisms has been explored in several studies [36,37]. Indeed, both OCT 3/4 and Sox2 are directly involved in Wnt and Notch signaling pathway, contributing to self-renewal and resistance of LSCs [36,38].

The role of SOX2 and OCT 3/4 as prognostic markers in the diagnosis and relapse of ALL has been highlighted in a recent studies, in agreement with previous research focused on AML specimens [39,40]. High levels of these markers are predictors of shorter overall survival (OS) and DFS in both ALL and AML patients and are associated with abnormal karyotypes, contributing to therapy resistance and higher risk stratification [40]. In CML, their reduction can decrease the LSCs population and sensitize these cells to targeted therapies [41,42]. Although the biological understanding of these processes is still limited in the field of leukaemia, they deserve further investigations. Targeting these pathways in combination with conventional therapies could improve treatment outcomes.

Furthermore, the identification of low levels of ROS in the pool of alive cells after treatment represents an important relevance in association with SC genes increase.

Indeed, SOX2 and OCT 3/4 play a crucial role in maintaining low ROS levels by regulating antioxidant defences, reprogramming metabolism, and suppressing pro-oxidative pathways [27,43]. This capability supports the survival and persistence of stem-like cells, protecting them against chemotherapy-induced oxidative damage, thereby contributing to drug resistance and relapse. Disrupting the ability of SOX2 and OCT 3/4 to regulate ROS levels could sensitize LSCs to oxidative stress and therapy, offering a potential treatment strategy. Through an untargeted analysis of the metabolites characterizing the surviving and the corresponding total cell population after drug treatment, we were able to identify several metabolites and pathways traceable to LSCs. Among these, glutathione and ubiquinone biosynthesis emerged as particularly important [44,45]. Their contribution to protecting cells from oxidative stress is critical for LSCs, as they help to maintain low ROS levels, supporting the quiescent state of LSCs, and preventing DNA damage. L-Palmitoylcarnitine and 3-Hydroxybutyrylcarnitine were identified as key metabolites involved in beta-oxidation and fatty acid catabolism (FAO), essential processes that sustain the energy needs of LSCs [46,47].

Additionally, AMP, an indicator of low energy status, is well known in the context of LSCs for its ability to activate AMPK (AMP activated protein kinase), which is crucial for maintaining the metabolic and energetic homeostasis of LSCs [48,49]. TCA cycle is often active in LSCs that rely on oxidative phosphorylation for energy production and focusing on this dependency could represent a promising approach to eliminate OXPHOS-dependent LSCs [50,51]. Finally, the significant number of pathways related to amino acid metabolism confirmed their importance in maintaining the stem-like characteristics of LSCs by supporting energy production, redox balance, self-renewal, and resistance to therapy [52,53].

Exploring the regulation of these metabolic markers could provide valuable targets for the development of clinical therapies aimed at contributing to the eradication of LSCs. Subsequently, thanks to an in-silico analysis of available datasets on two specific group of cells, K562 sensitive and resistant to Imatinib, we observed that among the most activated transcription factors in the resistant group there is Sox2, even if the different phenotypes that they assume do not match perfectly with LSCs characteristics, suggesting that cells can probably activate a program with some intermediate traits. Finally, the ChEA performed on CD34+ cells derived from CML patients after in vitro Imatinib treatment suggest important findings: only a few lists of deregulated genes emerge between the two specimens (not treated and Imatinib treatment), and the presence of Sox2, Nanog, and OCT 3/4 make our assumptions even more meaningful since they are validated on a tool of human samples [25,28]. Interestingly, the present ChEA analysis asserts that CML CD34+ cells after treatment display a potentially selective activation of TP53. The activation of p53 reflects the ability of cells to induce downstream TP53 effectors like BAX, NOXA, and BID and to disrupt anti-apoptotic proteins such as Mcl-1 and Bcl-2, favoring the induction of apoptosis after TKI treatment [54,55]. On the other hand, TP53 seems to be highly expressed in LSC, and, for this reason, developing strategies aimed at regulating orthogonal pathways, such as those associated with p53, could enhance the activity of TKI against CML stem cells [56,57].

## 5. Conclusions

All these evidences, including our results, suggest that several mechanisms significantly contribute to resistance of LSCs [58,59]. In our case, targeting Sox2, OCT 3/4 and their downstream pathways may offer potential therapeutic strategies to overcome resistance and improve treatment of CML patients. Moreover, further research is needed to fully understand the complex regulatory mechanisms involving SC features in LSC and their implications in treatment, prognosis, and in guiding different patients’ behavior. This should include shedding light on individuals that discontinue TKI therapy and experience CML recurrence and others that do not could contribute to optimizing the best therapeutic strategies.

Finally, both SOX2 and OCT3/4 could serve as potential markers useful for disease monitoring, relapse identification, and stratification of patients.

## Data Availability

All the data supporting the conclusions of this article will be made available by the authors upon request.
